# Contact-free trans-pars-planar illumination enables snapshot fundus camera for nonmydriatic wide field photography

**DOI:** 10.1038/s41598-018-27112-x

**Published:** 2018-06-08

**Authors:** Benquan Wang, Devrim Toslak, Minhaj Nur Alam, R. V. Paul Chan, Xincheng Yao

**Affiliations:** 1 Department of Bioengineering, University of Illinois at Chicago, Chicago, IL USA; 2 Department of Ophthalmology and Visual Sciences, University of Illinois at Chicago, Chicago, IL USA; 3 Center for Global Health, College of Medicine, University of Illinois at Chicago, Chicago, IL USA

## Abstract

In conventional fundus photography, trans-pupillary illumination delivers illuminating light to the interior of the eye through the peripheral area of the pupil, and only the central part of the pupil can be used for collecting imaging light. Therefore, the field of view of conventional fundus cameras is limited, and pupil dilation is required for evaluating the retinal periphery which is frequently affected by diabetic retinopathy (DR), retinopathy of prematurity (ROP), and other chorioretinal conditions. We report here a nonmydriatic wide field fundus camera employing trans-pars-planar illumination which delivers illuminating light through the pars plana, an area outside of the pupil. Trans-pars-planar illumination frees the entire pupil for imaging purpose only, and thus wide field fundus photography can be readily achieved with less pupil dilation. For proof-of-concept testing, using all off-the-shelf components a prototype instrument that can achieve 90° fundus view coverage in single-shot fundus images, without the need of pharmacologic pupil dilation was demonstrated.

## Introduction

Wide field fundus photography is desirable for screening, diagnosis, and treatment evaluation of diabetic retinopathy (DR)^1,2^, retinopathy of prematurity (ROP)^3,4^ and other eye diseases that can produce morphological abnormalities at peripheral areas of the retina. Traditional fundus cameras employ trans-pupillary illumination, i.e., a donut-shaped illumination pattern projected to the peripheral area of the pupil. After passing through the pupil, the light diverges and illuminates the posterior of the eye^5^. To illuminate the retina homogenously, the diameter and divergence of the illumination pattern on the pupil plane must be carefully adjusted, requiring careful design and sophisticated construction of the optical imaging system^6,7^. According to ISO 10940:2009^8^, external-angle is commonly used to specify field of view (FOV) in traditional fundus cameras. However, eye-angle has been recently adopted to determine the FOV in wide-field fundus imagers, such as a Retcam (Natus Medical Inc., Pleasanton, CA), Optos (Optos Inc., Marlborough, MA), etc. In order to avoid unnecessary confusion, we provide both external- and eye-angle numbers in the following discussion. Traditional fundus cameras provide 30°–45° external-angle (45°–67.5° eye-angle) FOV^9^. Additional challenges with trans-pupillary illumination include glare caused by light reflection from the cornea and crystalline lens^5,7^, and the requirement of adequate pupil dilation for wide field examination. Pharmacologic pupil dilation may make patients suffer from light glare and focusing difficulty for hours and even days in some cases.

Trans-scleral illumination has been proposed as one alternative illumination method to achieve wide field fundus examination not requiring pharmacologic pupil dilation^10,11^. Trans-scleral illumination delivers the illumination light from the region outside of the pupil, and thus can increase the available FOV for fundus photography (see Methods: Trans-pars-planar illumination). Panoret-1000™ employed trans-scleral illumination to image the retina from the optic disc to the ora serrata in a single-shot image^12^. However, clinical deployment of trans-scleral illumination was not successful, and the product Panoret-1000™ is no longer commercially available. Clinical deployment of trans-scleral illumination failed due to several limiting factors. First, the employed contact-mode imaging was not favorable for patients. Direct contact of the illuminating and imaging parts to the eyeball might produce potential inflammation, contamination, and abrasion to the sclera and cornea. Second, it was difficult to operate the system to obtain good retinal images. In Panoret-1000™, the digital camera and light illuminator were apart from each other. To capture a retinal image, one hand was used to operate the camera, and the other hand was used to adjust the light illuminator. The simultaneous need of both hands for imaging operation made the device very difficult to use. Moreover, the image quality was highly dependent on the illumination location. Without one objective, automated method for quickly optimizing location of the illuminator, it may take too long to train a photographer, and the ratio of readable retinal images was poor.

We recently demonstrated trans-palpebral illumination for wide-angle fundus photography^13^. Using a fiber illuminator contacting the eyelid, the trans-palpebral illumination excluded the possibility of eyeball inflammation, contamination, and abrasion associated with trans-scleral illumination. Without the need for pharmacologic pupil dilation, we have demonstrated nonmydriatic wide field fundus photography using a smartphone-based prototype^13^. However, contact-free imaging is desirable for easy-to-use clinical deployments.

In this article, contact-free trans-pars-planar illumination for wide field fundus photography is reported. Instead of using a light illuminator contacting the eyelid (trans-palpebral illumination)^13^ or sclera (trans-scleral illumination)^10,11^ trans-pars-planar illumination is totally contact-free to project illuminating light through the pars plana. Without the complications of contact optics and pupil dilation, the trans-pars-planar illumination promises a low-cost, easy-to-use, wide field fundus photography to advance routine examination of DR, ROP and other eye diseases that are known to produce morphological changes of the retinal periphery.

## Results

### Fundus photography with trans-pars-planar illumination

Using all off-the-shelf components, we constructed the prototype camera for proof-of-concept validation of trans-pars-planar illumination. Without the need of pharmacologic pupil dilation, a 60° external-angle (90° eye-angle) fundus view coverage was achieved in single-shot fundus images.

**Figure 1:**
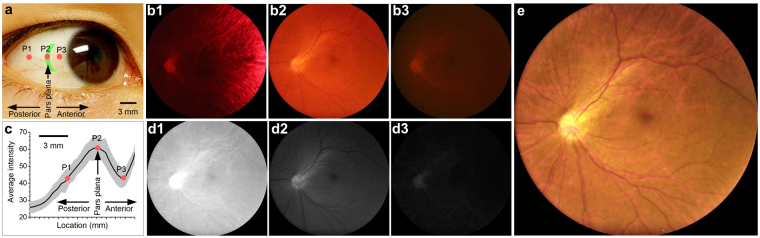
Representative fundus images with illumination at different locations. (**a**) Illustration of different illumination locations. (**b**) Fundus images acquired at different illumination locations. b1-b3 were acquired at corresponding locations P1-P3 in panel a. (**c**) Average intensity of fundus images collected with constant power illumination delivered through different locations. The curve is an average of 5 trials from one subject. Gray shadow shows standard deviation. P1-P3 corresponds to images b1-b3. (**d**) Red, green and blue channels of the fundus image b2. (**e**) Normalized fundus image b2, with digital compensation of red and green channel intensities. Macula, optic disc, nerve fiber bundles and blood vessels could be clearly identified.

As shown in Fig. 1a, one arc-shaped visible light pattern was used for trans-pars-planar illumination. Figure 1b shows representative fundus images collected with illumination delivered through areas posterior (Fig. 1b1), center (Fig. 1b2) and anterior (Fig. 1b3), respectively, to the pars plana. For all of these three images, the camera was set to exposure time 1 s, with ISO 3200 and white balance color temperature 3200 K. It was observed that the image quality was sensitive to illumination location. By pointing illumination pattern to the posterior of pars plana (P1 in Fig. 1a), choroidal vasculatures were observed in the fundus image dominated by red color (Fig. 1b1). By moving illumination pattern to the center of pars plana (P2 in Fig. 1a), retinal vasculatures, the optic disc, and the macula were unambiguously observed (Fig. 1b2). As localizing the illumination pattern to the anterior of pars plana (P3 in Fig. 1a), the image was too dim to reveal details of fundus structures (Fig. 1b3). In order to quantify the location dependent efficiency of light illumination, average pixel intensities of individual images, which were collected with illumination pattern scanned from the posterior sclera to the limbus at a step interval of ~0.4 mm, were illustrated in Fig. 1c.

All images in Fig. 1b were red predominated because of the superior penetration capability of long (e.g., red) wavelength light, compared to short (e.g. green and blue) wavelength light. For the image Fig. 1b2, the average intensity of red channel was 4 and 16 times higher than that of green and blue channels, respectively (Fig. 1d). In order to enhance the visualization of retinal structures, red and green channels were digitally balanced in Fig. 1e. Given the absence of blue light in the light source in the prototype instrument (see Methods: Experimental setup), the blue channel was ignored to reconstruct the enhanced image Fig. 1e. In Fig. 1e, macula and optic disc were clearly observed, and individual blood vessels were unambiguously identified. Moreover, nerve fiber bundles could also be observed as stripped patterns coming from the optic disc.

### Quantitative analysis of wide field fundus image

Quantitative analysis of fundus images is essential for objective and automated classification of eye diseases^14^. In order to verify the potential feasibility of using the trans-pars-planar illumination based fundus camera for quantitative imaging, we explored automated classification of arteries and veins, quantitative analysis of blood vessel diameter and tortuosity, and arteriolar-to-venular diameter ratio (AVR). It is known that retinopathy can affect arteries and veins differently. For example, some studies have shown that in ROP the increase in arterial tortuosity is more significant than that of veins^15^, and in DR the diameter of arteries decrease and diameter of veins increase^16,17^. Therefore, separate analysis of arteries and veins can provide improved sensitivity for quantitative fundus image analysis and classification.

**Figure 2:**
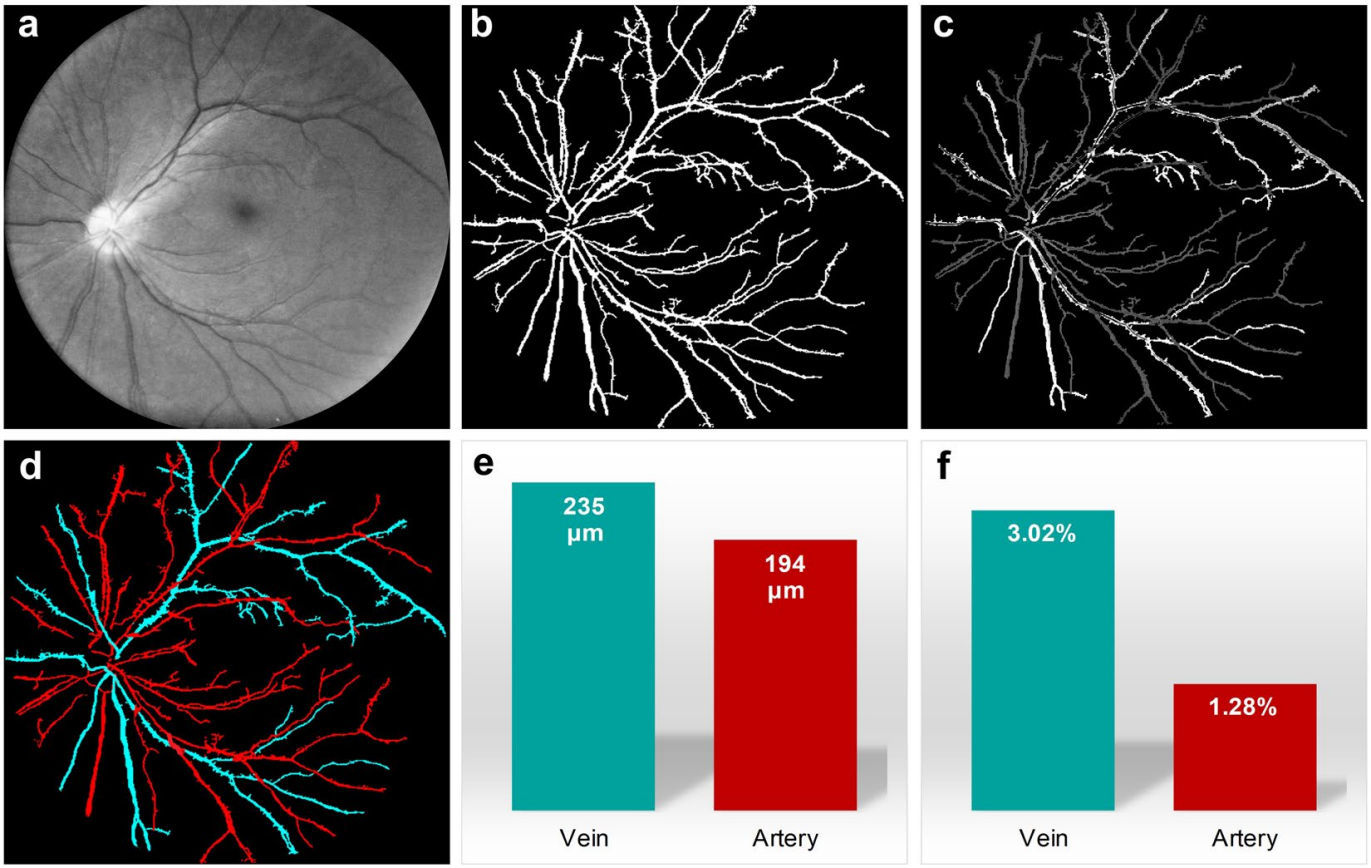
Automated arteries and veins classification of the wide field fundus image shown in Fig. 1b2. (**a**) The green channel of the fundus image in Fig. 1b2. Same image as Fig. 1d2 with brightness increased for better visualization. (**b**) Segmented blood vessel map using the green channel. (**c**) Optical density ratio map of red and green channels. (**d**) Arteries (red) and veins (cyan) classified by automated algorithm. (**e**) Average artery and vein diameters. (**f**) Average artery and vein tortuosity.

Figure 2 illustrates basic procedures of automated classification of retinal arteries and veins. Technical details are explained in Methods section. First, red and green channels were separated from a color fundus image (Fig. 1b2). Second, the green channel was used to segment individual blood vessels in Fig. 2a to reconstruct the blood vessel map (Fig. 2b). Third, the optical density ratio (ODR) between red and green channels was calculated^18^. As shown in Fig. 2c, arteries showed lower ODR value than veins. Fourth, a brightness threshold was applied in Fig. 2c to separate arteries and veins (Fig. 2d). The automated classification reasonably matches manual classification of arteries and veins. Figure 2e shows average diameters of arteries and veins. The AVR thus could be calculated as AVR = 194 μm/235 μm = 0.8, which is within normal range (0.54–0.82) reported in previous publication^19^. Figure 2f shows calculated artery and vein tortuosity.

### Comparative evaluation between the lab prototype nonmydriatic instrument and a clinical mydriatic fundus camera

**Figure 3:**
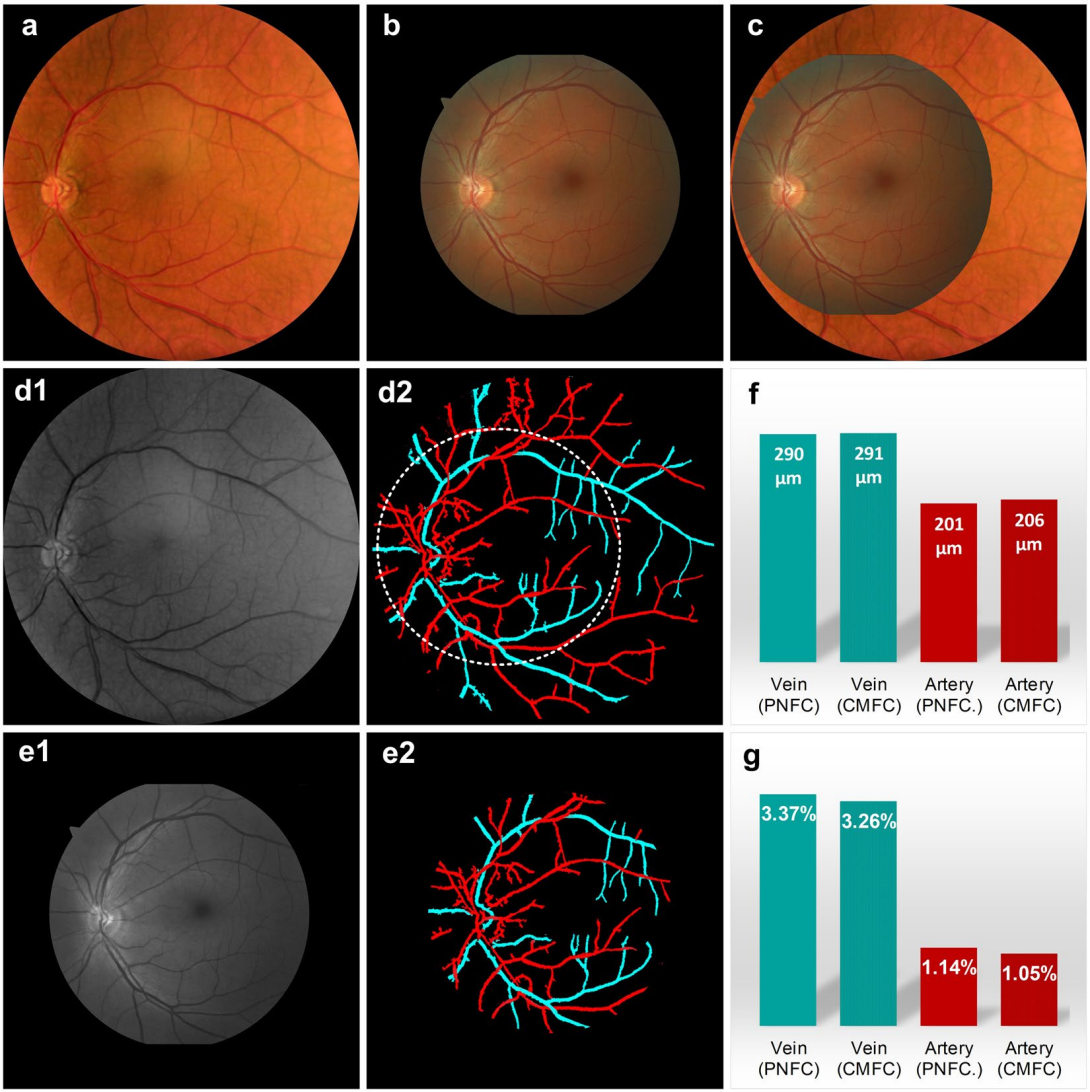
Comparative evaluation between the prototype nonmydriatic instrument and a clinical mydriatic fundus camera. (**a**) Fundus image captured with the prototype nonmydriatic fundus camera with 90° FOV (eye-angle, i.e., 60° external-angle). (**b**) Fundus image captured from the same subject using a clinical mydriatic fundus camera (Cirrus Photo 800, Zeiss), which has a FOV of 67.5° (eye-angle, i.e., 45° external-angle). (**c**) Overlap of images (**a**) and (**b**) for FOV comparison. (**d**) The green channel (d1) and classified arteries (red) and veins (cyan) (d2) of fundus image (**a**). (**e**) The green channel (e1) and classified arteries (red) and veins (cyan) (e2) of fundus image (**b**). (**f**) Average artery (red) and vein (cyan) diameters in the images of Fig. 3d2 (dashed white circle area) and Fig. 3e2. (**g**) Average artery (red) and vein (cyan) tortuosity in the images of Fig. 3d2 (dashed white circle area) and Fig. 3e2. PNFC: prototype nonmydriatic fundus camera; CMFC: clinical mydriatic fundus camera.

A clinical mydriatic fundus camera (Cirrus Photo 800, Zeiss) was used for comparative imaging to evaluate the image quality of the trans-pars-planar illumination based nonmydriatic fundus photography. Figure 3c shows overlapping illustration of the images shown in Fig. 3a,b. The clinical mydriatic camera has a FOV of 45° external-angle (67.5° eye-angle). The expanded FOV (60° external-angle, i.e., 90° eye-angle) of the nonmydriatic prototype instrument was directly observed (Fig. 3c). Fundus images captured with two systems revealed similar blood vessels, macula and optic disc. Particularly, the enhanced green channel (Fig. 3d1,e1) and artery-vein classification (Fig. 3d2,e2) show almost exactly the same retinal vasculatures in the overlapping area. Quantitative analysis of vessel diameter (Fig. 3f) and tortuosity (Fig. 3g) of the overlapping area further confirmed the great agreement between the nonmydriatic lab prototype instrument and the clinical mydriatic fundus camera.

## Discussion

A contact-free trans-pars-planar illumination for snapshot, wide field fundus photography without pharmacologic pupil dilation was demonstrated. In conventional fundus cameras with trans-scleral illumination, both illumination path and imaging path share the pupil. Typically, the illuminating light is delivered through the periphery of the pupil. In order to minimize the effect of reflections from the cornea and crystalline lens on fundus image^5,7^, the imaging light is typically collected through the central pupil only. Therefore, the available FOV of conventional fundus camera is congenitally limited, and pupil dilation is frequently required for fundus examination of the retinal periphery. Sophisticated system design, with delicate optical devices, were mandatory to balance the pupil usages for illumination light delivery and imaging light collection, making traditional fundus cameras complex and expensive. By freeing the entire pupil for collecting imaging light only, the demonstrated trans-pars-planar illumination dramatically simplified the complexity of optical system required for wide filed fundus photography. Moreover, the trans-pars-planar illumination eliminates all contact parts in previously demonstrated trans-scleral^12^ and trans-palpebral^13^ illumination for wide filed fundus photography. Therefore, totally contact-free trans-pars-planar illumination promises next generation low-cost, ultra-wide field, nonmydriatic, snapshot fundus cameras, which will foster clinical deployment of wide filed fundus photography to enable better ROP management, early DR detection, and improved accuracy in predicting DR progression and diabetic macular edema (DME) development, etc.^20,21^.

Using fundus images acquired with the prototype camera, the feasibility of automated classification of arteries and veins, quantitative analysis of blood vessel diameter, tortuosity, and AVR were validated. Because different eye diseases can affect arteries and veins differently^15–17^, separate analysis of arteries and veins can provide improved sensitivity for quantitative fundus image analysis and classification. Comparative evaluation was conducted using a clinical mydriatic fundus camera to demonstrate the potential of using the nonmydriatic trans-pars-planar illumination for clinical application. Moreover, we have recently demonstrated the feasibility of using smartphone for trans-scleral illumination based fundus photography with single-shot view up to 152°^13^. Unique combination of the contact-free trans-pars-planar illumination, low-cost smartphone technology, quantitative image analysis, and widely available internet technology promises a low-cost, ultra-wide field, nonmydriatic fundus camera to enable affordable telemedicine to reduce health disparities in rural and underserved areas, where both experienced ophthalmologists and expensive devices are limited.

While trans-pars-planar illumination provides an easy solution for wide field fundus photography, the image quality is highly dependent on illumination delivery location. In other words, accurate identification of pars plana is important. In our current prototype, the illumination position was manually controlled through a mechanical translation stage. In principle, automatic identification of pars plana can be achieved by dynamic monitoring of overall brightness of individual digital images as shown in Fig. 1c.

We are aware of different color efficiencies for trans-pars-planar illumination. It is known that light attenuation due to absorption and scattering is wavelength dependent. Because the transmission of long wavelength light is much higher than short wavelength light, we chose a LED that had strong short wavelength emission (yellow and green color) and weak long wavelength emission (red color) (see Methods: Experimental setup) for trans-pars-planar illumination. However, the fundus images were still red predominated. It is well established that details of retinal vasculatures are mainly reflected in short wavelength images. In order to produce retinal blood vessel enhanced images, we have demonstrated the feasibility of digitally normalizing intensity values of individual color channels (Fig. 1e). However, the effective dynamic ranges of red, green, and blue channels can be different for trans-pars-planar illumination with a single light source. For example, when the red channel is close to saturation level (i.e. 255 for an 8-bit camera), the green channel may only have light level ~60. In other words, the effective dynamic range of the green channel is only ~1/4 provided by the camera sensor. In our next generation prototype, we propose to integrate three separate light sources of different colors (red, green, blue: RGB) into the illuminator to enable individual R/G/B power controls to compensate for color difference of light efficiency of ocular tissues, and thus to maximize useful dynamic range of the digital camera.

One second exposure time was used for collecting the fundus image in Fig. 1. We are aware that prolonged exposure time could blur fundus images due to involuntary eye movements. We are currently testing flash light sources to pursue improved quality of fundus images. Using increased light power, we expect to control the exposure time to millisecond level to minimize the effect of eye movements on fundus image.

In conclusion, contact-free trans-pars-planar illumination for nonmydriatic, snapshot, wide field fundus photography was demonstrated. Using all off-the-shelf components, a simple and low-cost prototype instrument was constructed to validate a 90° fundus view in single-shot images, without the need of pharmacologic pupil dilation. The fundus images provided sufficient quality to allow computer-aided classification of arteries and veins, and quantitative analysis of blood vessel diameter, tortuosity, and AVR. Further development of the trans-pars-planar illumination based fundus camera will foster clinical deployment and telemedicine of using wide filed fundus photography for better disease screening, diagnosis, and treatment management of DR, ROP, DME, and other chorioretinal conditions.

## Methods

### Trans-pars-planar illumination

**Figure 4:**
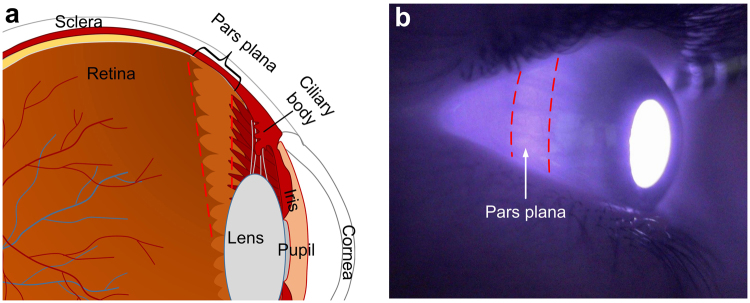
Schematic (**a**) and photographic (**b**) illustrations of pars plana. (**a**) Schematic cartoon showing the cross-section of the human eye. Pars plana is the area labeled between the dashed red lines. (**b**) Near infrared photograph showing the location of pars plan, marked by dashed red lines.

Figure 4 shows the anatomy and location of the pars plana. The pars plana is the smooth, posterior part of the ciliary body (Fig. 4a). It is a ~4 mm wide band located ~3–4 mm posterior to limbus^22,23^. The pars plana lacks muscle, blood vessels and pigmentation^23^, thus is more transparent than other area of the sclera, making it a good location for delivering light into the eye. Figure 4b shows an infrared image of the eyeball. The eyeball was illuminated with infrared light (850 nm) at the other side using a fiber that touched eyelid. As shown in Fig. 4b, the brightness of the pars plana was higher than other scleral areas because of its high transmittance.

**Figure 5:**
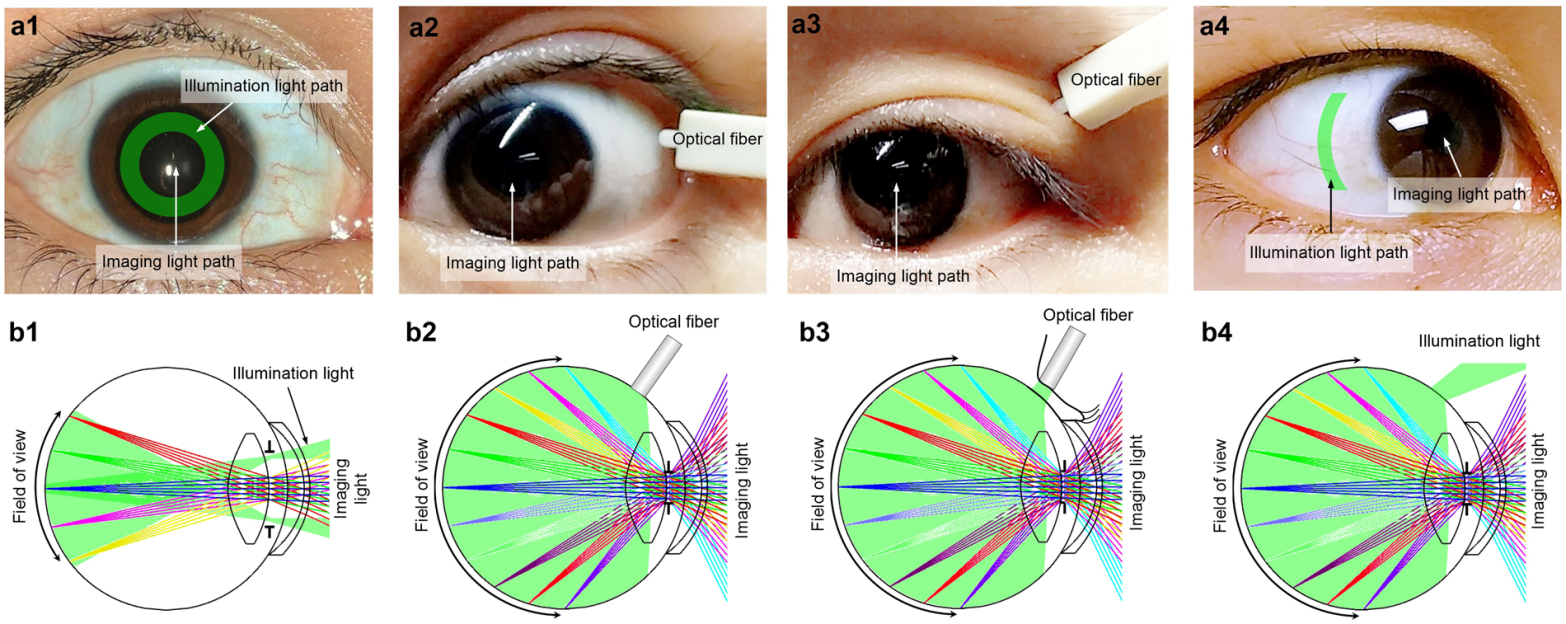
Schematic illustration of different illumination schemes for retinal imaging. (a1–a4) show illumination and imaging light paths of trans-pupillary illumination (a1), trans-scleral illumination (a2), trans-palpebral illumination (a3) and trans-pars-planar illumination (a4). (b1–b4) illustrate available FOVs with trans-pupillary illumination (b1), trans-scleral illumination (b2), trans-palpebral illumination (b3) and trans-pars-planar illumination (b4). The T shapes in b1 – b4 represent the pupil.

Figure 5 shows schematic illustration of trans-pars-planar illumination in comparison with other illumination schemes. In conventional fundus cameras, illumination and imaging light paths are spatially separated to avoid reflection from cornea and crystalline lens, i.e., the peripheral area of the pupil is used for illumination and the central area is used for imaging, to achieve reflection-free fundus imaging. As shown in Fig. 5a1, a donut shaped illumination pattern is projected to the pupil plane. The illumination light diverges after passing the pupil and illuminates the posterior area in the eye (Fig. 5b1). As shown in Fig. 5b1, such configuration requires a large pupil so that illumination and imaging light paths do not overlap on the cornea and crystalline lens surfaces. Pupil dilation is frequently required for wide field fundus photography. Figure 5a2,b2 show trans-scleral illumination to achieve nonmydriatic wide field fundus imaging. An optical fiber in contact with the sclera can be used to deliver illumination light into the back of the eye^10,11^. The trans-scleral illumination frees the entire pupil for imaging, and thus nonmydriatic wide field fundus imaging is possible. However, the contact mode illumination is not favorable, and thus it failed clinical deployment. We recently reported a prototype of trans-palpebral illumination fundus camera, which delivered illumination light through the eyelid, without direct contact to the sclera (Fig. 5a3,b3). However, because of the absorption of the eyelid and sclera, the illumination light efficiency was relatively low. To increase the illumination efficiency, we propose here to develop contact-free trans-pars-planar illumination (Fig. 5a4,b4). As shown in Fig. 5a4, an arc-shaped illumination pattern, which matches the shape of the pars plana, was projected to the pars plana without physical contact between the illuminator and the sclera. After passing the pars plana, the illumination light was diffused and illuminated the intraocular area homogenously (Fig. 5b4). Since corneal reflection was intrinsically eliminated and the entire pupil was used for imaging, wide field fundus photography was made possible without pharmacologic pupil dilation (Fig. 5b4).

### Experimental setup

**Figure 6:**
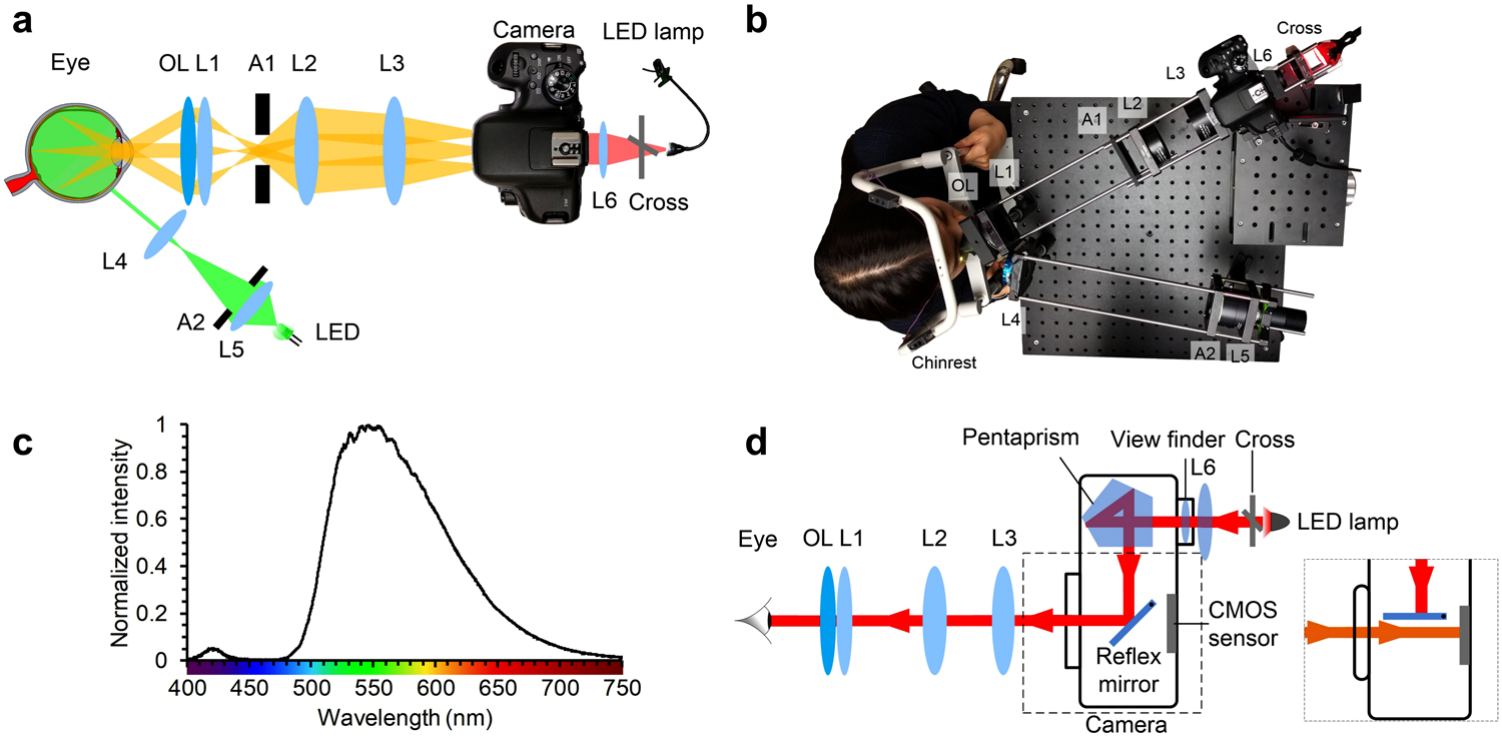
Experimental setup. (**a**) Schematic diagram of the system showing the illumination light path, imaging light path, and fixation target placed behind the viewfinder. OL: ophthalmic lens; L1-L6: lenses; A1: circle aperture; A2: annular aperture. (**b**) Picture showing fundus image was being taken using our prototype system. (**c**) Spectrum of the LED used for illumination. (**d**) Schematic diagram showing the light path of the fixation target. Inset shows when capturing the fundus image, the reflex mirror temporarily flips up and the light from the eye reaches the CMOS sensor.

Figure 6a shows the system diagram and Fig. 6b shows a photograph of the lab prototype camera. A 565 nm LED (Fig. 6c. M565L3, Thorlabs) was selected as the light source for color fundus imaging. Light from the LED was collected by a lens and then passed through an arc-shaped aperture. A lens was used to image the aperture onto the sclera to form an arc-shaped illumination pattern. The illumination aperture was carefully designed to closely match the shape of the pars plana. The end of the illuminating arm that was close to eye could be manually moved in a horizontal direction by a translation stage to precisely deliver illumination light to the pars plana. Light passing through the pars plana was diffused and illuminated the intraocular area homogenously. A 22D ophthalmic lens (Volk Optical, Inc.) was used to collect light coming out of the pupil. Three off-the-shelf lenses (Thorlabs) were placed after the ophthalmic lens to relay the fundus image onto the CMOS sensor of a digital single-lens reflex camera (EOS Rebel T6i, Canon Inc.). An aperture was placed at the pupil conjugate plane to restrict effective imaging pupil size to 2.5 mm for best imaging resolution^24,25^, as well as to reject scattering light from the sclera. A lens was positioned behind the camera viewfinder and a cross that was illuminated by an LED lamp was placed in front of the lens to serve as a fixation target, so that the testing subjects could fix their eyes by looking into the camera through the lenses and look at the cross. A single-shot fundus image could be easily acquired by pressing the camera shutter button.

### Fixation target

In conventional fundus cameras, a beam splitter can be used to split light paths so that a fixation target could be implemented. However, a beam splitter wastes a fraction of the light from the retina. In our prototype system, there was no beam splitter required due to the single reflex feature of the camera. As shown in Fig. 6d, the reflex mirror reflected the light from the fixation target to the eye so that the subject could see the cross. When shutter was pressed, the reflex mirror temporarily flipped up and light coming out from the eye reached the CMOS sensor and formed a fundus image (Fig. 6d inset).

### Human subject

This study was approved by the Institutional Review Board of the University of Illinois at Chicago and was in compliance with the ethical standards stated in the Declaration of Helsinki. Images shown were captured from one healthy Asian female subject and one health Turkish male subject with informed consent. No discomfort or vision impairment was reported by the subjects after fundus photos being taken.

### Light safety

Potential photochemical and thermal hazards of the retina were carefully evaluated. There is no retina present at the pars planar area. However, it is possible that the light passes through sclera and illuminates the retina. The thickness of the sclera is ~0.5 mm^26^. The transmission of the sclera in visible wavelength is 10–30%^27^. To be conservative, 30% was used for calculation. According to the ISO 15007-2:2007 standard, a maximum of 10 J/cm^2^ weighted irradiance is allowed on the retina^28^ without photochemical hazard concern. The weighted irradiance was calculated using the photochemical hazard weighting function provided in the standard. For the proof-of-concept experiment, the weighted irradiance on the sclera was calculated to be 0.5 mW, the area of the arc-shaped light was 13 mm^2^. For the worst case estimation, we assumed all illumination light directly expose to the retinal area behind the illuminated sclera area. Therefore, the maximum allowed exposure time is

1 $${t}_{\max }=\frac{10\,{\rm{J}}\,/{{\rm{cm}}}^{2}}{0.5\,{\rm{mW}}\times 30 \% /13\,{{\rm{mm}}}^{2}}=2.4\,{\rm{h}}$$

If the illumination light accidently fell into the pupil, the illuminated area on retina was estimated to be >9 mm^2^. Thus the maximum allowed exposure time through the pupil is >30 minutes. For thermal hazard, the maximum weighted power intensity allowed on the sclera without thermal hazard concern is 700 mW/cm^2 ^^28^. The calculated weighted power intensity was 230 mW/cm^2^, which was more than three times lower than the maximum limit. Therefore, there was no thermal hazard concern.

### Quantitative analysis of wide field fundus image

To identify arteries and veins, the blood vessel map was first segmented using the green channel of the fundus image. The green channel (Fig. 2a) was used because the contrast between blood vessels and background was high^29^. The segmentation algorithm was based on the traditional matched filtering method first developed in 1989^29^. Briefly, 2D Gaussian kernels that matched blood vessel profiles were used. Kernels of 12 different orientations and 10 different sizes were used to cover all blood vessel directions and diameters. After subtracting their means, each kernel was used to convolve the green channel fundus image and a total of 120 convolved images were generated. Among the 120 convolved images, the maximum intensity projection was used as the final enhanced blood vessel image. Top-hat filtering was used to correct uneven illumination of enhanced blood vessel image, then global thresholding was applied to generate segmented blood vessel map (Fig. 2b). ODR image was then calculated using equation (R-G)/G, where R was the red channel image and G was the green channel image^18^. The ODR image was then multiplied by the blood vessel map (Fig. 2c). Blood vessels were automatically identified as artery or vein based on the fact that ODR of artery was lower than vein^18^, and diameter of artery was smaller than accompanying vein^30^. Details of blood vessel tortuosity and diameter quantification have been reported in previous publication^14^.

### Data availability

The datasets generated and analyzed during the current study are available from the corresponding author on reasonable request.
